# 1244. Antimicrobial Stewardship Knowledge, Attitudes, and Practices (KAP) Among Nurses

**DOI:** 10.1093/ofid/ofad500.1084

**Published:** 2023-11-27

**Authors:** Jillian E Hayes, Reinaldo Perez, Ali R Winters, Rebekah W Moehring, Rebekah Wrenn

**Affiliations:** Duke University Hospital, Durham, North Carolina; Duke University, Durham, North Carolina; Duke Private Diagnostic Clinics, Durham, North Carolina; Duke University, Durham, North Carolina; Duke University, Durham, North Carolina

## Abstract

**Background:**

Nurses are underutilized members of the antimicrobial stewardship (AS) team. Understanding knowledge, attitudes, and practices (KAP) is essential to designing effective AS initiatives. While KAP surveys have been performed among a variety of provider types, less is known about KAP among nurses. We describe KAP pertaining to AS among nurses at a large, academic hospital.

**Methods:**

An electronic, 24-question KAP survey was designed by the AS team with relevant nursing and infection prevention champions. Nine knowledge-based questions about antimicrobials focused on 4 domains: beta-lactams first in sepsis, intravenous to oral transition, urinary tract infections, and penicillin allergies. Voluntary responses were collected via Qualtrics from January 9, 2023-March 31, 2023. Surveys were distributed by email link, with reminders to enhance survey response. Incentives included a chance to win a meal voucher.

**Results:**

85 complete survey responses were received. The majority of respondents worked on dayshift (69%) in a staff nurse role (66%). Discrepancies were seen in perceptions of issues of stewardship nationally and locally. While nurses felt antimicrobial resistance and harm caused by antimicrobials are high nationally (92 and 69%, respectively), this was not as commonly felt to be true on a local level (35% and 26%). The majority (87%) agreed or strongly agreed that nurses’ responsibility includes promoting appropriate antimicrobial use. When assessing knowledge of AS domains, reported confidence in performance was discordant with actual performance and differed by domain (Figure 1). Respondents were largely unfamiliar with common AS reference resources (73-74%) and were more likely to consult an ID pharmacist (81%), ID physician (78%) or infection prevention team member (78%) with questions.

**Figure:**
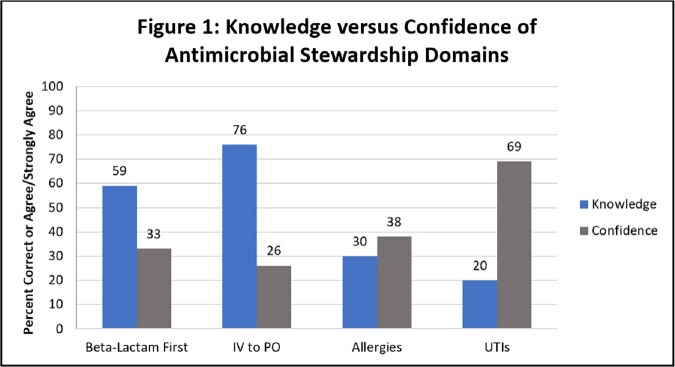

**Conclusion:**

Nurses recognized AS as an important part of their professional responsibilities, but perceived AS opportunities to be a national rather than local need. Differing patterns in knowledge vs. confidence among topic domains provides key information to then design relevant, sustainable, nurse-driven antimicrobial stewardship initiatives.

**Disclosures:**

**Rebekah W. Moehring, MD, MPH, FIDSA, FSHEA**, UpToDate, Inc.: Author Royalties

